# Comparison of the Effects of Nintedanib and Pirfenidone on Pulmonary Function Test Parameters and Radiological Findings in Patients with Idiopathic Pulmonary Fibrosis: A Real-Life Study

**DOI:** 10.3390/medicina61020283

**Published:** 2025-02-06

**Authors:** Olcay Aycicek, Serra Keskin, Muhammed Haciosmanoglu, Funda Oztuna, Yilmaz Bulbul, Tevfik Ozlu

**Affiliations:** Department of Chest Disease, Faculty of Medicine, Karadeniz Technical University, Trabzon 61100, Turkey; serrakeskin@ktu.edu.tr (S.K.); muhammedhaciosmanoglu@ktu.edu.tr (M.H.); foztuna@yahoo.com (F.O.); bulbulyilmaz@yahoo.com (Y.B.); ozlutevfik@yahoo.com (T.O.)

**Keywords:** idiopathic pulmonary fibrosis, pirfenidone, nintedanib

## Abstract

*Background and Objectives:* The aim of our study is to compare the effects of pirfenidone and nintedanib on lung function and radiologic findings in Idiopathic Pulmonary Fibrosis and to identify which drug is more appropriate for which patient group. *Materials and Methods:* The data of patients who were treated in our department for at least one year between 1 January 2010 and 31 December 2022 and who were started on pirfenidone or nintedanib treatment with the diagnosis of Idiopathic Pulmonary Fibrosis were retrospectively reviewed. The patients were divided into two groups—the nintedanib and pirfenidone groups—and both groups were compared in terms of progression in lung function tests (changes in FEV1, FVC, 6 MWT and DLCO values at the 3rd, 6th, 9th and 12th months compared to baseline values) and radiological findings (the presence of progression in findings such as ground-glass opacity, reticulation, honeycomb and traction bronchiectasis) within 1 year after diagnosis. *Results:* The study included 109 patients. The number of patients treated with pirfenidone (IPF patients) was 82 (75.2%) and the number of patients treated with nintedanib was 27 (24.8%). When the PFT values at 3, 6, 9 and 12 months were compared with the baseline values in both groups, there was no statistically significant difference in any parameter between the two groups. No significant difference was found in terms of radiological progression at the end of 1 year in both groups. *Conclusions:* The results of our study show that pirfenidone and nintedanib are equivalent in their effectiveness in preventing disease progression in patients with IPF.

## 1. Introduction

Idiopathic Pulmonary Fibrosis (IPF) is one of the interstitial lung diseases (ILDs), and is a progressive lung disease of unknown cause that is pathologically compatible with the usual interstitial pneumonia (UIP) pattern [1]. Despite being the most prevalent form of interstitial lung disease, its overall incidence is low. The annual incidence in the United States of America has been found to be between 1.2 and 76.4 per 100,000 [2,3]. The most common population is male smokers over the age of 65 [4]. IPF is a difficult disease to treat and treatment alternatives are limited. Pirfenidone and nintedanib are two antifibrotic drugs indicated for the treatment of IPF patients. There are studies showing that both drugs reduce decreases in FVC (Forced Vital Capacity), which is a determinant of the severity of restriction in IPF; decrease exacerbation frequency and hospitalizations; and increase survival rate [5,6,7].

Interstitial lung diseases other than IPF can also progress to fibrosis. However, antifibrotic therapy is indicated when fibrosis is progressive, and this is called progressive pulmonary fibrosis (PPF). To diagnose PPF, at least two of the following criteria must be present within the last year: worsening of respiratory symptoms, physiological evidence of disease progression (absolute decrease in FVC ≥ 5% or absolute decrease in DLCO ≥ 10% (corrected for Hb) or radiological evidence of disease progression (increase in the extent or severity of traction bronchiectasis and bronchiolectasis, or the development of new ground-glass opacities or new reticulations with traction bronchiectasis) [8]. Recent studies show that antifibrotic drugs with proven efficacy in IPF may also be effective in progressive pulmonary fibrosis [9,10]. Although the evidence regarding pirfenidone is not clear in this regard, there are important studies showing the positive effects of nintedanib on pulmonary function tests in this group of patients [6].

There have been many studies on these two drugs separately in both IPF and non-IPF patients, but there are only a few studies comparing these two drugs with each other. The criteria for which drug is preferred in diagnosed patients are not clear. The aim of our study is to compare the effects of pirfenidone and nintedanib on lung function and radiologic findings in IPF.

## 2. Materials and Methods

The data of patients who started pirfenidone or nintedanib treatment with the diagnosis of Idiopathic Pulmonary Fibrosis according to an Official ATS/ERS/JRS/ALAT Clinical Practice guideline and treated for at least one year in our department between 1 January 2010 and 31 December 2022 were retrospectively analyzed. The included patients had not previously used steroids or similar drugs for lung disease. The study was initiated after the approval of the local ethics committee. Since our study was a retrospective study, informed consent was not required for participation according to local legislation and was not obtained.

File information, pulmonary function test parameters and radiological data, gender, age and comorbidity information of all patients were obtained from the hospital database. Pulmonary function tests were performed using VIASYS, SensorMedics, Yorba Linda, CA, USA Healthcare Vmax ENCORE 22 (until 2021) and COSMED, Rome, Italy, Quark 2021 model devices. Patients were divided into two groups: the nintedanib and pirfenidone groups. The drugs were administered at a dose of 4 × 600 mg in the pirfenidone group and 2 × 150 mg in the nintedanib group, and both groups were compared in terms of progression in pulmonary function tests and radiologic findings within 1 year of diagnosis. For this purpose, pulmonary function test parameters (FVC (mL-%), FEV1 (mL-%)) and 6-min walk test and DLCO values at baseline and the 3rd, 6th, 9th and 12th months were compared. Then, the differences in FVC, FEV1, 6MWT and DLCO values at 3, 6, 9 and 12 months from the baseline values at the time of diagnosis were analyzed and these differences were compared between the two groups. Thorax CT findings at the time of diagnosis were compared between the two groups in terms of the rates of ground-glass opacity, reticulation, honeycomb and traction bronchiectasis and the presence of progression in these findings within 1 year.

Patients whose radiologic and PFT data at initial presentation were not available, patients whose treatment was discontinued before 1 year due to side effects or unresponsiveness or death, and patients who were switched between the two drugs before 1 year had elapsed were excluded from the study.

### Statistical Analysis

The Kolmogorov–Smirnov test was used to test the normal distribution of the continuous variables. The data characterized by a normal distribution are expressed as mean ± standard deviation. Student’s *t*-test was used for the comparison of the data which had a normal distribution. The Mann–Whitney U test was used for the comparison of the non-normally distributed data. The discrete variables were compared using the Chi-squared test. *p* < 0.05 was considered to be statistically significant. The data were analyzed using the SPSS statistical software (version 13.01, serial number 9069728, SPSS Inc., Chicago, IL, USA).

## 3. Results

After the exclusion of patients who did not meet the inclusion criteria, 109 patients were included in the study. There were no deaths within 1 year among the patients included in the study. The gender distribution of patients was as follows: 87 (79.8%) male and 22 (20.2%) female. The mean age was 69.90 ± 8.65 years in men and 68.64 ± 10.19 years in women (*p* = 0.55).

The number of patients receiving pirfenidone treatment was 82 (75.2%) and the number of patients receiving nintedanib treatment was 27 (24.8%). The demographic characteristics of the patients are given in Table 1.

**Figure 1:**
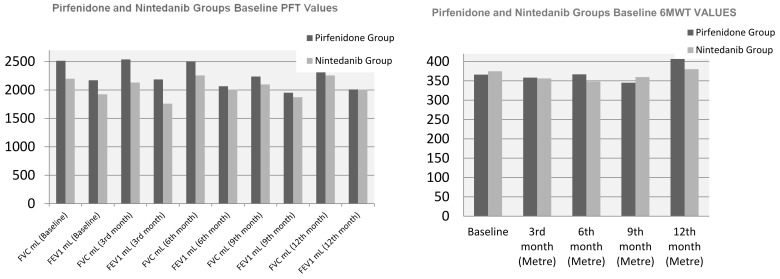
Comparison of PFT and 6MWT values in pirfenidone and nintedanib groups.

Figure 1 shows the comparison of the baseline, 3-month, 6-month, 9-month and 12-month PFT and 6MWT parameters of the pirfenidone and nintedanib groups. There is no statistically significant difference between the two groups.

When the SFT values and 6-min walk test values at the 3rd, 6th, 9th and 12th months in both groups were compared with baseline values, no statistically significant difference was found in any parameter between the two groups. Due to the lack of DLCO test data, the difference in response between the groups could only be made according to the 12th month. The 12th-month mean DLCO values were 44.27 ± 14.16 in the pirfenidone group and 35.71 ± 7.99 in the nintedanib group, and although the difference between these two values was statistically significant (*p* = 0.01), the rates of change from baseline were similar in both groups (*p* = 0.743) (Table 2).

No statistically significant difference was found when oxygen saturations before and after the 6-min walk test performed at the time of admission and at the 3rd, 6th, 9th and 12th months were compared in both groups (Table 3).

The pirfenidone and nintedanib groups were compared with respect to the presence of ground-glass opacity, increased reticulation, honeycombing and traction bronchiectasis, which are the most common radiological findings in interstitial lung diseases at the time of diagnosis. There was no statistically significant difference in the incidence of these radiological findings between the two groups (Table 4).

When both groups were evaluated in terms of the presence of radiological progression after 1 year, progression was observed in 11 (13.4%) patients in thee pirfenidone group and in 5 (18.5%) patients in the nintedanib group, and there was no statistically significant difference between the two groups in this respect (*p* = 0.538).

Table 5 presents the details of patients who were discontinued or switched due to disease progression or the emergence of adverse effects necessitating a change in treatment. Among patients receiving nintedanib, only two (7%) of the patients experienced severe diarrhea to the extent that they required treatment discontinuation. In the pirfenidone group, liver enzymes increased in one (1.2%) patient and photosensitivity developed in two (2.4%) patients, but treatment discontinuation or dose reduction were not required.

## 4. Discussion

Our study is an observational real-life study comparing 1-year treatment outcomes in patients with IPF treated with pirfenidone and nintedanib.

Pirfenidone and nintedanib are two antifibrotic drugs that have been approved and proven to be effective in the treatment of Idiopathic Pulmonary Fibrosis and are used worldwide. Many clinical studies have shown that these two drugs slow down the rate of decline in functional residual capacity, decrease the number of exacerbations and improve survival rates in IPF [5,11,12]. Nintedanib has been shown to be effective in interstitial lung diseases in non-IPF as well as IPF patients. Lung involvement is common in systemic sclerosis and is the most important cause of mortality. In patients with ILDs associated with systemic sclerosis, the annual rate of decline in FVC was found to be lower in patients treated with nintedanib than placebo [13]. Studies have also shown that nintedanib significantly reduced the annual rate of decline in FVC compared to placebo in patients with lung disease with progressive pulmonary fibrosis other than scleroderma [14]. There are insufficient data on the efficacy of pirfenidone in non-IPF ILDs [6]. There is no doubt that the two drugs are effective in IPF, but the number of studies comparing the efficacy of nintedanib and pirfenidone in slowing or preventing the progression of the disease is very limited.

Bargagli et al. conducted a retrospective study investigating the efficacy of these two drugs in IPF patients. In this study, FVC, FEV1, TLC and DLCO values at baseline, 6 and 12 months were compared in 82 IPF patients diagnosed in the last 12 months who were treated with pirfenidone (n = 52) and nintedanib (n = 30). No significant difference was found in the percentages of FVC, FEV1, TLC and DLCO at baseline between patients treated with the two drugs (*p* = 0.59, *p* = 0.37, *p* = 0.21, *p* = 0.48, respectively). At 6-month follow-up, there was no significant difference in the % of predicted values of FVC, FEV1, DLCO or TLC between patients treated with pirfenidone and nintedanib (*p* = 0.54, *p* = 0.38, *p* = 0.76, *p* = 0.31, respectively). Due to the late approval of nintedanib in Italy, only the pirfenidone group was able to complete the 12-month period. When the pirfenidone group was evaluated within itself, no statistical difference was found between the baseline ad 6th- and 12th-month values of the mentioned parameters. The study showed that treatment with both drugs had similar rates of decrease in FVC at 6-month follow-up [15].

In another study by Cerri et al., 142 IPF patients were divided into three groups: pirfenidone-treated (n = 78), nintedanib-treated (n = 28) and a control group that received no treatment (n = 36), and patients were compared at 6, 12 and 24 months for factors such as pulmonary function test, arterial blood gas, liver function test, side effects and treatment adherence. After adjustment for baseline differences in FVC, a statistically significant reduction in this parameter was observed over the time period in the control group compared with the treated groups (*p* = 0.0053), while no significant difference was observed between the pirfenidone and nintedanib groups over the 24-month time course. After adjustment for baseline differences in DLCO, a statistically significant decrease in this parameter was observed in untreated patients (*p* = 0.037), but no statistically significant difference was observed between the pirfenidone and nintedanib groups and DLCO remained stable over the 24-month time course [16].

In a retrospective study by Cameli et al. analyzing 10 years of data, 263 IPF patients were included in the study. Of these patients, 139 were treated with pirfenidone and 124 with nintedanib. The median survival time of the patients was 1224 days, and there was no difference between the pirfenidone and nintedanib groups in terms of survival time and the time until FVC decreased by more than 10% (*p* = 0.8786 and *p* = 0.1677, respectively). At the end of the 1-year period, a smaller decrease in DLCO values was found in the nintedanib group compared to the other group (*p* = 0.016), but this difference closed as the follow-up period increased. The lesser decrease in DLCO in the nintedanib group was attributed to the antiangiogenic property of nintedanib [17].

In a study comparing 840 patients treated with pirfenidone and 713 patients treated with nintedanib in terms of mortality, hospitalization and care costs, both groups were similar in terms of two-year all-cause mortality (HR: 0.90, 95% CI: 0.76; 1.07), one-year all-cause mortality (HR: 1.09, 95% CI: 0.95; 1.25) and respiratory-related hospitalizations (HR: 0.89, 95% CI: 0.72; 1.08). Also, no significant difference was observed between the two groups in terms of total (EUR- 807, 95% CI: EUR- 2977; EUR 1220) and respiratory-related costs (EUR- 1282, 95% CI: EUR- 3423; EUR 534). However, this study did not analyze the change in pulmonary function and was very different from our study [18].

In another study, patients with IPF diagnosed with pirfenidone or nintedanib were followed up, and at the end of 12 months, the FVC values of patients treated with nintedanib were found to be higher than those of the pirfenidone group (mean difference, 106 mL; 95% CI, 34–178). However, it was observed that this difference narrowed by the end of the 24-month follow-up [19].

In our study, we divided 109 patients with IPF into two groups: the pirfenidone and nintedanib groups. As expected, the majority of the patients were male [N = 87 (79.8%)], the mean age was 69.64 ± 8.94 years and there was no statistical difference between the two groups. No significant difference was found in the baseline, 3rd month, 6th month, 9th and 12th month PFT and 6MWT parameter values of patients in the pirfenidone and nintedanib groups. When the differences in the 3rd, 6th, 9th and 12th month PFT values compared to baseline in both groups were compared, no statistically significant difference was found in any parameter between the two groups. In our study, unlike the studies cited above, we did not compare the absolute PFT values of both groups at the 3rd, 6th, 9th and 12th months in our study. By comparing the changes in the values of both groups at the specified months with the basal values, we aimed to more accurately reveal whether the changes were favourable or unfavourable. As a result, we did not find any statistically significant difference in any parameter between the two groups when the differences in the 3rd, 6th, 9th and 12th month PFT values compared to baseline were compared in both groups. Another difference of our study from the cited studies is that we compared both groups in terms of radiological progression. The rates of ground-glass opacity, increased reticulation, traction bronchiectasis and honeycombing, which are the most frequently observed findings in IPF, were statistically similar in the pirfenidone and nintedanib groups at the time of diagnosis. When both groups were evaluated for the presence of radiological progression after 1 year, progression was observed in 11 (13.4%) patients in the pirfenidone group and in 5 (18.5%) patients in the nintedanib group, and there was no statistically significant difference between the two groups in this respect (*p* = 0.538).

In almost all previous studies comparing the efficacy of pirfenidone and nintedanib in IPF patients, the efficacies of these two drugs were found to be equivalent. In line with the literature, our study showed that the effects of pirfenidone and nintedanib on pulmonary function test parameters, 6MWT and radiologic progression were similar at 1-year follow-up.

The limitations of our study are that it was a retrospective study and the number of patients was small.

We hope to reach a larger patient population by investigating the efficacy and safety of the two drugs in non-IPF fibrosis diseases as well as in IPF in prospective studies with a larger patient population.

## 5. Conclusions

The results of our study show that pirfenidone and nintedanib are equivalent in preventing worsening of pulmonary function and radiologic progression in patients with IPF.

## Figures and Tables

**Table 1:** Demographic characteristics of IPF patients.

	Pirfenidone Group	Nintedanib Group	Total
	n = 82 (75.2%)	n = 27 (24.8%)	n = 109 (100%)
**Gender**			
Male	66 (80.5%)	21 (77.8%)	87 (79.8%)
Female	16 (19.5%)	6 (22.2%)	22 (20.2%)
**Mean Age**	69.22 ± 9.47	70.93 ± 7.13	69.64 ± 8.94
**Comorbidity**			
Diabetes Mellitus	21 (25.6%)	5 (18.5%)	26 (23.8%)
Hypertension	35 (42.6%)	14 (51.8%)	49 (44.9%)
Cardiac Disease	28 (34.1%)	11 (40.7%)	39 (35.7%)
Neurological Disease	4 (4.87%)	3 (11.1%)	7 (6.42%)
Peripheral Artery Disease	2 (2.43%)	2 (7.40%)	4 (3.66%)
COPD	7 (8.53%)	1 (3.70%)	8 (7.33%)
Cancer	8 (9.75%)	3 (11.1%)	11 (10.0%)
Asthma	2 (2.43%)	2 (7.40%)	4 (3.66%)

**COPD:** Chronic obstructive pulmonary disease.

**Table 2:** Differences in PFT values compared to baseline in two groups.

	Pirfenidone Group N = 82 (75.2%)	Nintedanib Group N = 27 (24.8%)	
**Changes in PFT Values Compared to Baseline**	Mean ± Std. Deviation Mean (Min–Max)	Mean ± Std. Deviation Mean (Min–Max)	***p***
**FVC mL (3rd month)**	88.15 ± 489.46 50.00 (−920–1060)	153.75 ± 461.270 180.00 (−500–950)	0.694
**FEV1 mL (3rd month)**	147.86 ± 570.11 90.00 (−1260–2230)	145.00 ± 293.063 125.00 (−130–740)	0.970
**FVC mL (6th month)**	45.7143 ± 489.70 60.00 (−920.00–1040.00)	257.27 ± 246.58025 290.00 (−40.00–610.00)	0.164
**FEV1 mL (6th month)**	140.58 ± 713.33 30.00 (−870.00–2891.00)	177.27 ± 214.20042 170.00 (−80.00–660.00)	0.321
**FVC mL (9th month)**	235.92 ± 531.62 130.00 (−450.00–1850.00)	74.28 ± 371.07309 40.00 (−480.00–630.00)	0.594
**FEV1 mL (9th month)**	204.81 ± 442.14 90.00 (−570.00–1090.00)	60.00 ± 278.50793 40.00 (−440.00–430.00)	0.733
**FVC mL (12th month)**	200.00 ± 484.42 125.00 (−570–1420)	131.67 ± 339.35 70.00 (−370–750)	0.770
**FEV1 mL (12th month)**	215.48 ± 489.05 120.00 (−510–2250)	185.83 ± 278.12 155.00 (−210–620)	0.946
**6MWT metre (3rd month)**	−24.08 ± 120.96 17.00 (−340–102)	−21.00 ± 77.782 −21.00 (−76–34)	0.713
**6MWT metre (6th month)**	7.41 ± 86.15 8.50 (−187.00–136.00)	−97.50 ± 274.04 −0.50 (−476.00–215.00)	0.638
**6MWT metre (9th month)**	28.87 ± 103.65 30.50 (−187.00–153.00)	−68.00 ± 212.32 0.00 (−306.00–102.00)	0.406
**6MWT metre (12th month)**	−34.93 ± 115.54 −8.50 (−374–68)	−1.14 ± 242.53 110.00 (−527–170)	0.126
**DLCO mL/dk/mm (12th month)**	7.5 ± 10.91	8.6 ± 5.8	0.743

**PFT:** pulmonary function test, **FVC:** forced vital capacity, **FEV1:** forced expiratory volume, **6MWT:** *6-*min walk test, **DLCO:** diffusing capacity of the lungs for carbon monoxide.

**Table 3:** Comparison of SaO_2_ values before and after 6-min walk test.

	Initial SaO_2_ (%)			End of Test SaO_2_ (%)		
	Pirfenidone	Nintedanib	*p*	Pirfenidone	Nintedanib	*p*
**Bazal**	94.27 ± 2.34	95.11 ± 2.49	*0.204*	89.29 ± 6.22	90.94 ± 5.72	*0.332*
**3. month**	94.58 ± 3.05	92.50 ± 0.70	*0.371*	88.00 ± 7.17	95.00 ± 2.82	*0.210*
**6. month**	94.00 ± 3.41	94.83 ± 1.83	*0.587*	84.16 ± 9.70	88.50 ± 3.78	*0.313*
**9. month**	94.75 ± 2.54	94.00 ± 2.64	*0.690*	88.00 ± 3.81	90.33 ± 3.78	*0.389*
**12. month**	95.92 ± 1.75	94.28 ± 2.75	*0.120*	91.00 ± 4.4	86.57 ± 6.60	*0.091*

**Table 4:** Radiologic findings at diagnosis in IPF patients.

	Pirfenidone Group N = 82 (75.2%)	Nintedanib Group N = 27 (24.8%)	Total N = 109 (100%)	*p*
**Ground-Glass Opacification**	35 (43.2%)	15 (55.6%)	50 (46.3%)	*0.266*
**Reticulation**	71 (87.7%)	27 (100.0%)	98 (90.7%)	*0.63*
**Honeycombing**	62 (76.5%)	24 (88.9%)	86 (79.6%)	*0.147*
**Traction Bronchiectasis**	67 (82.7%)	25 (92.6%)	92 (85.2%)	*0.348*

**Table 5:** Progression and side effects after one year.

Initial Treatment	Treatment Duration (Month)	Reason	Outcome	Treatment Duration (Month)	Reason	Outcome
Pirfenidone	20	Progression	Stop	-	-	-
Pirfenidone	42	Progression	Stop	-	-	-
Pirfenidone	13	Progression	Nintedanib	6	Progression	Stop
Nintedanib	16	Diarrhea	Stop	-	-	-
Pirfenidone	20	Progression	Nintedanib	7	Progression	Stop
Nintedanib	47	Progression	Stop	-	-	-
Pirfenidone	12	Progression	Nintedanib	5	Diarrhea	Stop

## Data Availability

The corresponding author has all responsibility for the data, and can be contacted for the data.
